# Intensified modulation of the Pacific north equatorial current bifurcation by the southern annular mode since the early 1990s

**DOI:** 10.1038/s41598-022-25661-w

**Published:** 2022-12-08

**Authors:** Li-Chiao Wang, Yong-Fu Lin, Chau-Ron Wu

**Affiliations:** 1 Department of Atmospheric Sciences, National Central University, Taoyuan, Taiwan; 2 Department of Earth System Science, University of California Irvine, Irvine, CA USA; 3 Department of Earth Sciences, National Taiwan Normal University, Taipei, Taiwan; 4 Research Center for Environmental Changes, Academia Sinica, Taipei, Taiwan

**Keywords:** Physical oceanography, Physical oceanography

## Abstract

Long-term reanalysis data were used to assess inter-decadal to decadal modulations of the North Equatorial Current (NEC) bifurcation in the Pacific after the early 1990s. The wind stress curl anomaly (WSCA) in the region of 10° N–15° N and 160° E–170° E (C-BOX) had been found to excite Rossby waves and control NEC bifurcation along the Philippine coast. Our analysis revealed that the WSCA in the C-BOX has been remotely modulated by the Southern Annular Mode (SAM) since the early 1990s. It is shown that the SAM shifted to its positive phase at this transition and began strongly impacting the WSCA in the C-BOX and the NEC bifurcation. During the positive SAM phase after the early 1990s, strong climate variability occurred in the tropical to subtropical area of the North Pacific, with a clear footprint connected to the Antarctic region. Consistent with that finding, we determined that during the positive SAM phase, a dipole sea surface temperature pattern was generated in the South Pacific; this induced an atmospheric Rossby wave train in upper-level wind shear that propagated northward to the North Pacific. Such effects further enhanced downward motion and divergence at the surface, intensifying the easterlies in the equatorial area and the anticyclonic WSCA in the C-BOX. The anticyclonic WSCA in the C-BOX substantially excited downwelling oceanic Rossby waves at the surface, inducing an equatorward trend of NEC bifurcation after the early 1990s.

## Introduction

The westward-flowing North Equatorial Current (NEC) plays a prominent role in the tropical–subtropical circulation system of the Pacific Ocean^1–8^. As the NEC approaches the Philippine coast, it splits into two branches: the branch flowing northward feeds the Kuroshio Current, whereas the branch flowing southward forms the Mindanao Current. These western boundary currents eventually shift to an eastward direction and become the Kuroshio Extension to the north and the North Equatorial Countercurrent to the south, indicating that the NEC is essential for the development of the North Pacific subtropical and tropical gyres. Moreover, the NEC regulates heat, mass, and salt exchanges between the Kuroshio Current and the Mindanao Current^1,2^. Therefore, elucidation of the NEC bifurcation latitude (NECBL) variations is essential for exploring changes in low-latitude circulation and mid-latitude circulation.

Because there are limited long-term oceanic data, few studies have investigated the interannual variability of NEC bifurcation. One study suggested that the NECBL shifts northward 1 year after an El Niño event, whereas it shifts southward during La Niña events, based on the results of a numerical model^1^. That study also showed that NEC variations are governed by the basin-wide wind stress curl anomaly (WSCA). One year after El Niño, the positive WSCA intensifies and the zero wind stress curl (WSC) line shifts northward, affecting the location of NEC bifurcation. Kim et al.^2^ suggested that meridional displacement of the NEC bifurcation may result from westward-propagating upwelling (downwelling) Rossby waves driven by the central equatorial wind field and cyclonic (anticyclonic) WSCA in the western North Pacific during the mature phase of an El Niño (La Niña) event. A later study^3^ indicated that low-frequency variability in the NEC bifurcation cannot be fully represented from El Niño–Southern Oscillation (ENSO) index.

Recently, Wang et al.^9^ demonstrated that the NEC tends to have a more poleward bifurcation latitude when upwelling Rossby waves are stronger. They also found that the WSCA over an area of the central Pacific (10° N–15° N, 160° E–170° E, hereafter the C-BOX) where the NEC passes is tightly associated with the induction of Rossby waves and thereby impacts fluctuations in the NEC bifurcation. Their analysis demonstrated that the WSC in the C-BOX and the associated induction of Rossby waves are regulated jointly by the ENSO and Pacific Decadal Oscillation (PDO)^10,11^. They noted that Rossby waves are strongly enhanced when only one of the PDO and ENSO signals is in a strong positive phase^9^. Thus, the C-BOX appears to be deeply impacted by interannual to decadal climate variability over the Pacific region.

Considering that the C-BOX is situated over the North Pacific Subtropical Gyre (NPSG), variability in the NPSG is also strongly associated with variations in the WSC over the C-BOX region. Liu et al.^12^ examined the effects of the Southern annular mode (SAM) on the South China Sea monsoon; they found that the SAM influences climate variability over the NPSG area. Furthermore, the remote modulation effect of the SAM on East Asian monsoons over the NPSG has been confirmed^13^. Since the monsoons might have an impact on wind field of the NPSG region where the C-BOX is located in, we inferred that the SAM might play an important role on the C-BOX variability and the consequential NECBL fluctuations. In addition to the local climate modulation associated with the ENSO and the PDO^9^, remote inter-decadal to decadal climate variability driven by the SAM might also play an essential role on WSC variations over the C-BOX area, with substantial impacts on the generation of Rossby waves and meridional displacement of the NECBL.

## Results

### Relationship among wind field, Rossby waves, and the NECBL

**Figure 1:**
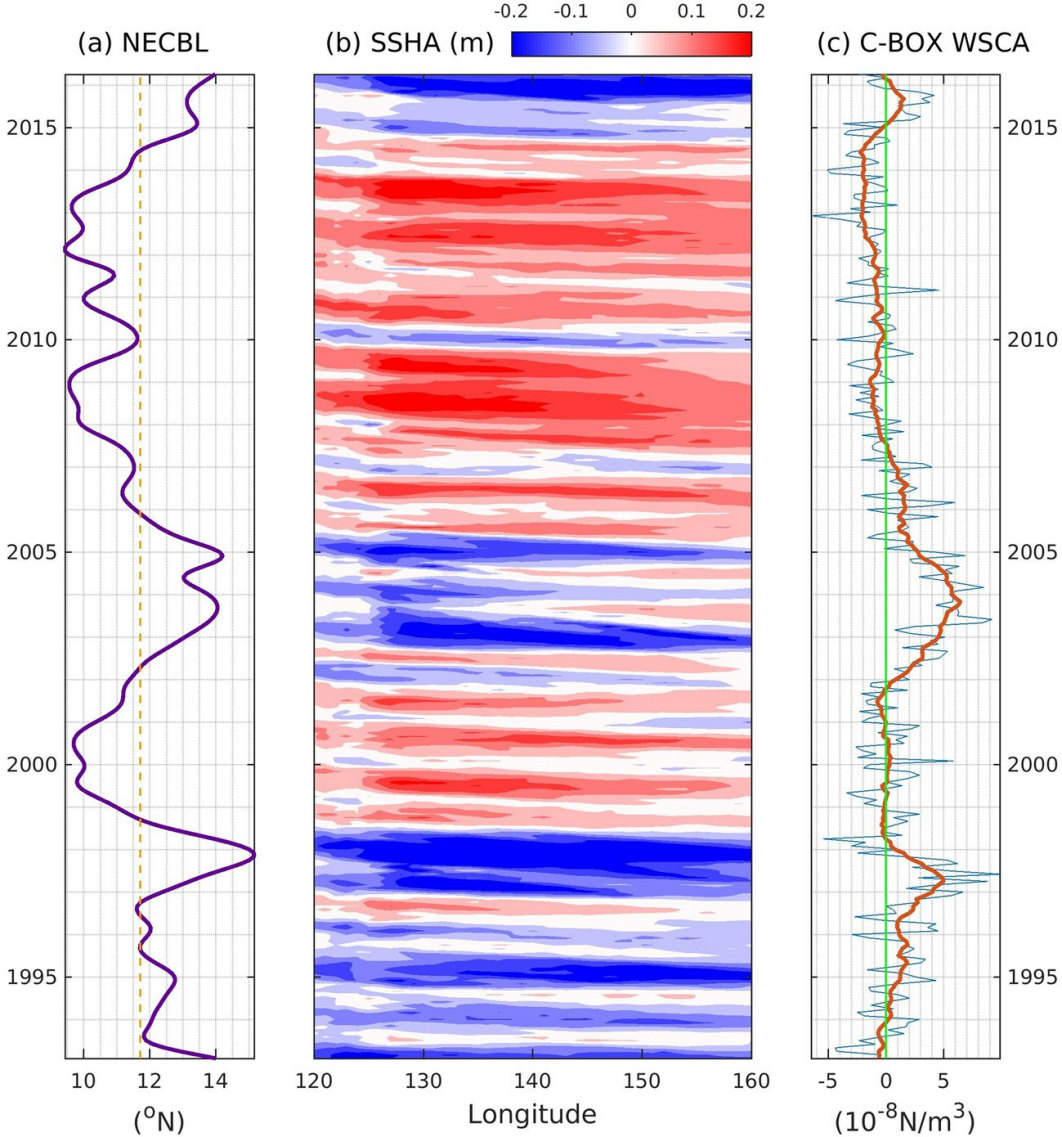
(**a**) Time series of the NEC bifurcation latitude derived from monthly sea surface height measured by satellite altimeter (unit: deg; data based on Fig. 2a of Qiu and Chen^3^). Orange line represents the mean value. (**b**) Sea surface height anomalies averaged over 10° N–15° N (unit: m) (**c**) The WSCA averaged over the C-BOX (10° N–15° N and 160°E–170° E, units: 10^–8^ N/m^3^). Data are averaged over the 10–15° N region. Red line represents the 13-month running mean.

**Figure 2:**
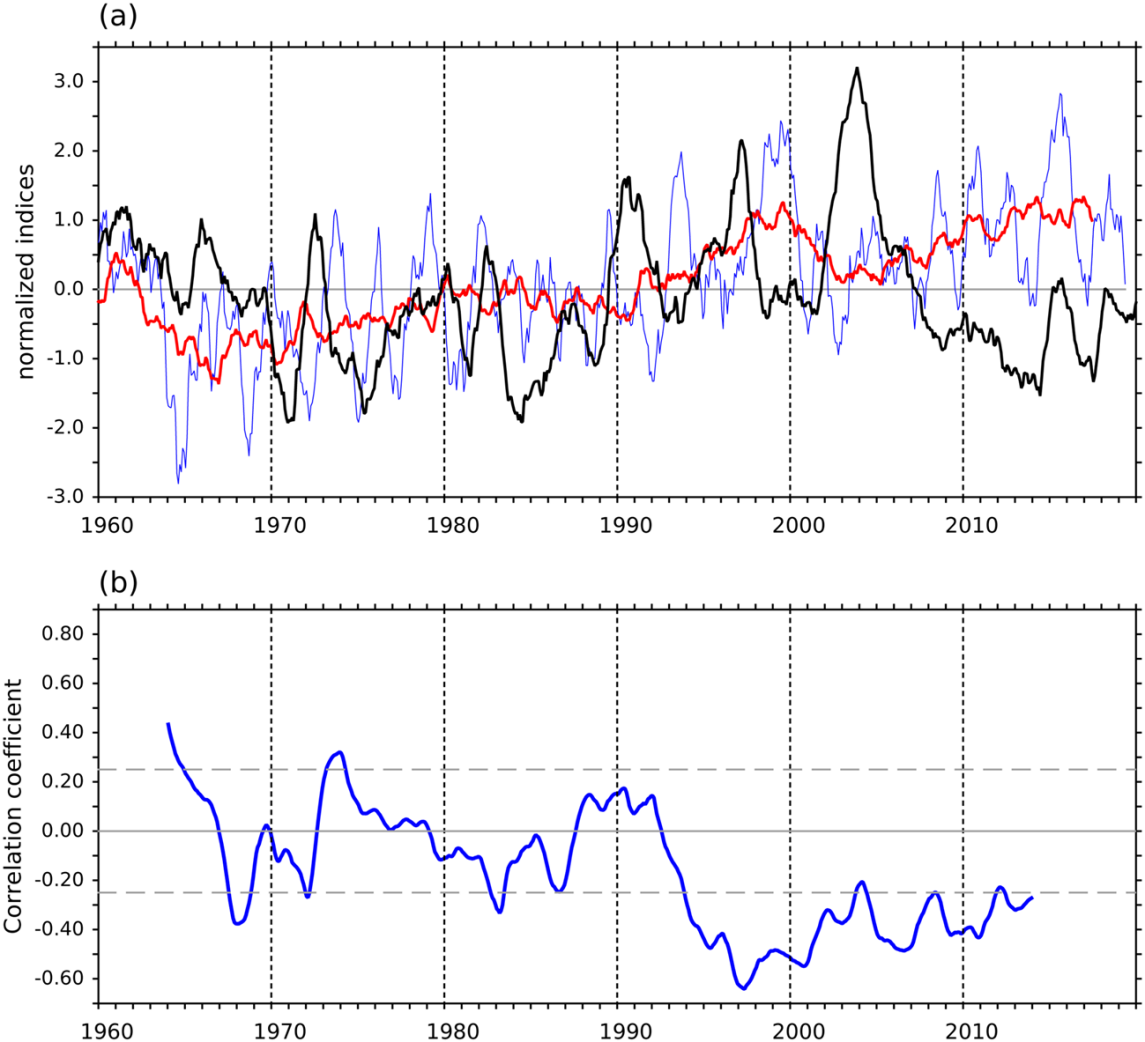
(**a**) Time series of normalized SAM index (blue line) and the WSCA in C-Box (black line) after 15-month running mean. The red line denotes the normalized SAM index after 61-month running mean. (**b**) 121-month running correlation between the SAM and the WSCA in C-BOX. All time series are calculated using 15-month running mean. Gray dash lines indicate statistically significance at the 90% confidence level determined by a Student’s t-test.

Previous studies^2,9^ indicated that the cyclonic (anticyclonic) WSCA in the central Pacific may excite westward-propagating upwelling (downwelling) Rossby waves, which could be identified via lower (higher) sea level height. These processes eventually result in a northward (southward) displacement of the NECBL along the Philippine coast. As demonstrated in Fig. 1, the NECBL was shifted poleward in 1997 and from 2003 to 2004. After 2004, the NECBL was shifted equatorward (Fig. 1a, data based on Fig. 2a in the work by Qiu and Chen^3^). Rossby wave activities in the area of the NEC fluctuation can be examined via the observed sea level height pattern averaged over 10° N–15° N. The sea level height exhibited a pattern that was consistently out of phase with the NECBL, which was negative during 1997 and 2003–2004 and remained positive after 2004 (Fig. 1b). This tendency shows that Rossby waves generated in the interior ocean are highly related to the meridional shift of the NECBL^1,2^. Furthermore, the WSCA in the C-BOX (10° N–15° N, 160° E–170° E) is strongly associated with the excitation of Rossby waves and subsequent migration of the NECBL. The WSCA in the C-BOX (10° N–15° N, 160° E–170° E) showed cyclonic patterns in 1995–1997 and 2002–2004, followed by frequent weak anticyclonic patterns after late 2007 (Fig. 1c). These features support the previous finding that cyclonic (anticyclonic) WSCA in the C-BOX generates westward-propagating upwelling (downwelling) Rossby waves, which subsequently induce poleward (equatorward) movement of the NEC bifurcation site. The correlation between the NECBL and the low-passed WSCA over C-BOX is 0.65, which is statistically significance at the 95% confidence level determined by a Student’s t-test. Therefore, the WSCA in the C-BOX can be used to identify variations in the NECBL^5^. Moreover, decadal variability can be observed through this series of processes. Because of the limited availability of satellite measurements of oceanic data, we used the WSCA in the C-BOX to explore the decadal modulation effect of the SAM on variations in the NECBL.

### Modulation effect of the SAM on wind variations in the C-BOX

In addition to local climatic variability, the NPSG is influenced by polar climatic variability through the SAM^12,13^. Therefore, the wind field in the C-BOX, which is located in the NPSG region, is strongly influenced by the SAM. Figure 2a shows the relationship between the WSCA in the C-BOX and the SAM climate index during 1960–2019. The correlation between the WSCA and the SAM index over that entire period was − 0.29. Nevertheless, decadal variability exists in the relationship between the SAM and the WSCA in the C-BOX. The SAM index had a weak correlation with the WSCA before the 1990s, and then became nearly out of phase with WSCA after 1995. A 10-year running-correlation analysis was conducted to further explore this relationship (Fig. 2b). The running-correlation between the SAM and the WSCA in the C-BOX oscillated between − 0.2 and 0.2 before the 1990s. After 1995, the SAM became highly negatively correlated with the WSCA in the C-BOX, reaching − 0.6 in 1997. Our results imply that the SAM might start to have an influence on the north Pacific from the early 1990s as it shifted from the negative to positive phase (the red line in Fig. 2a), and gradually came to impact the wind variations over the C-BOX region after 1995.

**Figure 3:**
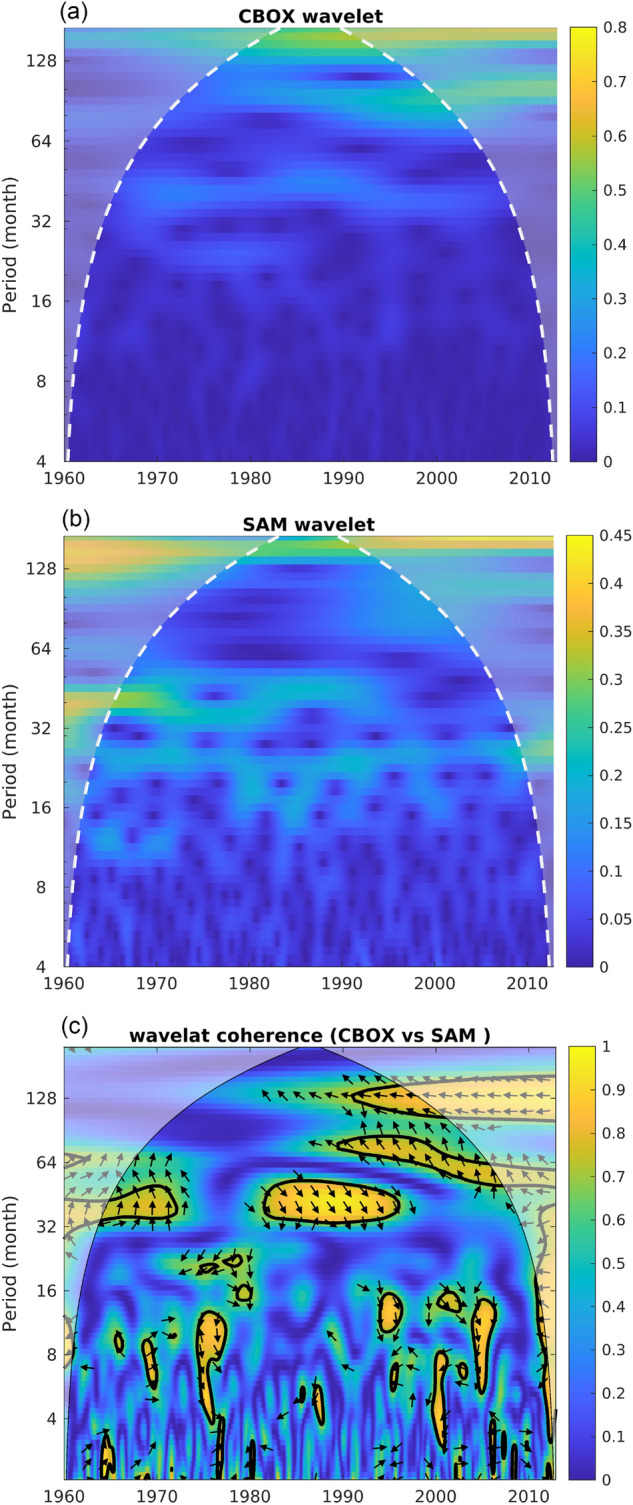
(**a**) Wavelet power spectrum of 61-month running mean of the WSCA in C-BOX. (**b**) Same as (a), but for 61-month running mean of the SAM index. White dash lines in (**a**) and (**b**) represent the cone of influence. (**c**) Squared wavelet coherence between 61-month running mean of the WSCA in the C-BOX and the SAM index. The contours denote the 95% confidence level against red noise. The arrows represent the relative phase relationship (in-phase pointing right; the SAM index leading WSCA in the C-BOX by 90° pointing straight down).

To further explain the C-BOX–SAM relationship, Fig. 3 shows the wave spectrum of the WSCA in the C-BOX (Fig. 3a) and the SAM index (Fig. 3b). In wavelet analysis of the WSCA in the C-BOX, a high power was assigned to the decadal variability of approximately 128 months, which is inside the cone of influence and has strengthened since the 1990s. In the wave spectrum of the SAM index, a high power of the decadal variability around 128 months is also present before 1970 and after 1990. The wavelet coherence analysis (Fig. 3c) further demonstrates that the decadal variability of the WSCA in the C-BOX after 1990 could be attributed to the SAM. The arrows pointing left around 128 months after 1990 also support that the WSCA in the C-BOX and the SAM index have a negative correlation (same as the result of Fig. 2b).

## Mechanism

**Figure 4:**
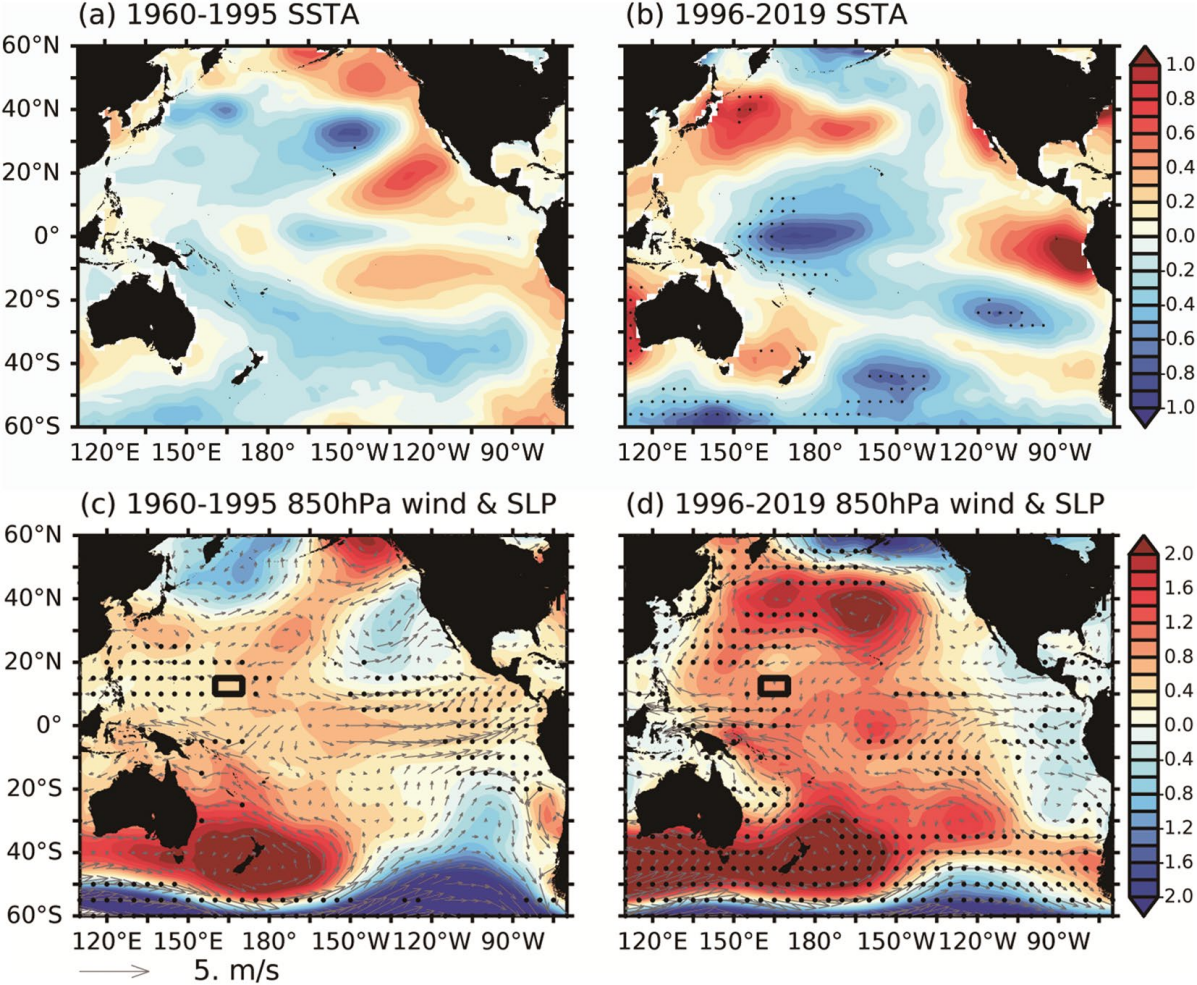
(**a**) Regression of 15-month running mean of SSTA (unit in °C) onto 15- month running mean of the SAM index during 1960–1995. (**b**) Same as (**a**), but for 1996–2019. (**c**) Same as (**a**), but for SLPA (shading, unit in hPa) and wind field anomalies (vectors, unit in m/s) at 850 hPa. (**d**) Same as (**c**), but for 1996–2019. Stippled area in all figures indicates statistically significance at the 95% confidence level determined by a Student’s t-test.

To clarify the decadal change in the Pacific climate after 1995, the oceanic and atmospheric structures in the Pacific were regressed onto the SAM index to contrast between two periods: 1960–1995 and 1996–2019 (Fig. 4). Compared with 1960–1995 (Fig. 4a,c), the period of 1996–2019 showed stronger regressions in both the oceanic and atmospheric fields (Fig. 4b,d). Because the SAM was positive following 1996, the regressions well represent the anomaly patterns. A cold sea surface temperature anomaly (SSTA) occurred in the southeastern Pacific and was extended to the central tropical region by the south-easterly wind anomalies via the wind-evaporation SST (WES) feedback. The entire temperate zone was occupied by a cold SSTA connected to the Antarctic area. This SSTA pattern reveals the footprint of the SAM, which is initiated in the polar area and expands northward to the entire Pacific region. More detailed analysis was conducted using the 850-hPa anomalous wind field and sea level pressure anomaly (SLPA). During the period of 1960–1995, the SLPA and atmospheric circulation were weak in the northern Pacific (Fig. 4c). In contrast, the period of 1996–2019 experienced an enhancement of SLPA and more apparent wind circulation pattern compared with 1960–1995, especially in the North Pacific. Strong easterlies occurred in the tropical area west of the dateline and enhanced the anticyclonic WSCA around the C-BOX (black rectangle), whereas strong westerlies were observed east of 150°W. These results demonstrate that active climate variability over the North Pacific after 1995 is strongly associated with modulation by the SAM. These regressed patterns confirmed that the SAM exerted a stronger control on the Pacific conditions from 1996–2019 than from 1960–1995. The impact of the SAM on C-BOX becomes much more stable since 1996 during its continuing positive phase.

**Figure 5:**
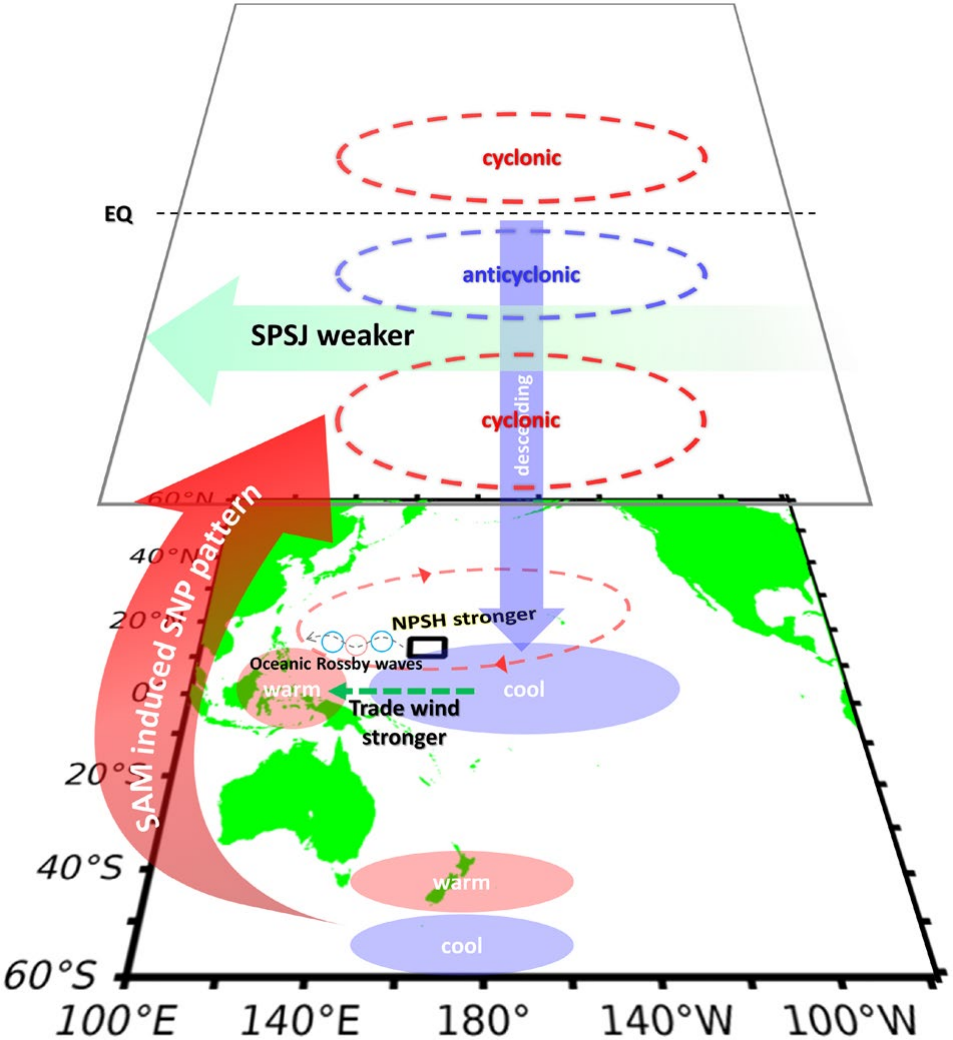
Diagram of the linkage between the Atlantic, South Pacific and North Pacific. After 1995, a positive SAM favors a positive Southern Pacific SST Dipole (SPD). Coordinated variation of the SPD induces a weaker Southern Pacific Subtropical Jet (SPSJ) and positive South–North Pacific teleconnection pattern (SNP) wave train with anomalous downward motion over the tropical central Pacific^12^, which strengthens mean surface easterlies over the western Pacific. The easterly wind would accumulate the warm SST in the warm pool of the tropical western Pacific to the west, then causing a warm SST in the eastern Indian Ocean and the Maritime Continent. The increased SST gradient further intensifies the Pacific trade winds and the associated North Pacific Subtropical High (NPSH)^14,15^. Red and blue ellipses indicate SNP teleconnection pattern. Black box indicates the C-BOX region.

A recent study provided insights into the impacts of the SAM on the NPSG^12^. Based on those findings, Fig. 5 illustrates the sequence of oceanic and atmospheric effects induced by the SAM, and then links them with wind variations in the C-BOX. First, the positive SAM signal after 1995 favors a dipole SST pattern at the surface in the South Pacific. This dipole SST pattern can modify the subtropical jets around Australia, inducing alternating anticyclonic and cyclonic shear in the Pacific Ocean. Anomalous wave activities resulting from upper-level divergence/convergence anomalies can further excite the wave train, which propagates northward to the North Pacific (Liu et al.^12^, see also Fig. S1). Upper-level convergence anomalies around 0°N–40°N enhance descending motions, causing a high-pressure zone and divergence wind field at the surface; these findings explain the SLPA and wind patterns shown in Fig. 4d. Therefore, the easterlies are strengthened west of the dateline, enhancing the warm SST in the western Pacific. This series of effects strengthens the pressure gradient between the cold tongue area and the warm pool area, enhancing the easterlies over the western tropical Pacific via a positive Bjerknes feedback. The increased SST gradient and the intensified trade winds further amplifies North Pacific Subtropical High (NPSH), which reinforces the anticyclone in the North Pacific and the anticyclonic WSCA in the C-BOX. Our results demonstrate that the SAM may be an important climate factor responsible for strengthening of the anticyclonic WSCA in the C-BOX after 1995. Under the modulation of positive SAM, the anticyclonic WSCA in the C-BOX enhances oceanic downwelling Rossby waves, eventually resulting in an equatorward shift of the NEC bifurcation.

## Conclusion

Previous research demonstrated that the NECBL shifts northward in the presence of strong upwelling Rossby waves^1,2^. The WSCA in the C-BOX has been strongly associated with the generation of Rossby waves and impacts on fluctuations in the NEC bifurcation. In the current study, we found that the SAM, which had minimal impact on the NPSG during its negative phase before 1990, shifted into a dominant role on variations of the WSCA in the C-BOX region beginning in 1995. The influence of the SAM on the WSCA in the C-BOX was confirmed through wavelet analysis. A strong signal with a 128-month period appears in the power spectra of both the SAM and the C-BOX wind field, suggesting a strong modulation effect of positive SAM on the C-BOX since the early 1990s.

To further explore the decadal variability in the influence of the SAM on the NPSG and particularly the C-BOX, we regressed oceanic and atmospheric structures onto the SAM index as and contrasted between two periods: 1960–1995 and 1996–2019. Compared with 1960–1995, the regression patterns of 1996–2019 showed much greater variability in the distributions of SSTA, SLPA, and wind patterns, suggesting that the SAM exerted a stronger control on the Pacific conditions from 1996–2019 than from 1960–1995. Strong easterlies occurred in the tropical area west of the dateline and enhanced the anticyclonic WSCA in the C-BOX (black rectangle). Our results also indicate that the strong climate variability in the NPSG and the C-BOX after 1995 is closely associated with modulation by the SAM. Based on the findings in a previous study^12^, the pathway through which the SAM controls the WSCA in the C-BOX can be discerned. Because the SAM became positive in the early 1990s, a dipole SST pattern was generated in the South Pacific. The associated upper-level cyclonic and anticyclonic shear induced a Rossby wave train that propagated northward and eventually induced upper-level convergence at 0°N–40°N, enhancing downward transport and divergence at the surface. This divergence strengthened the mean surface easterlies over the western Pacific, thereby increasing the warm SSTA in the eastern Indian Ocean and the Maritime Continent. Through the Bjerknes feedback, the increased SST gradient further intensified the surface easterlies and the associated NPSH^14,15^. The enhanced surface easterlies to the south of the C-BOX strengthened the anticyclonic wind stress curl in the C-BOX.

In summary, in the early 1990s as the SAM became positive, a series of air–sea interactions were induced that propagated the contribution of the SAM from the Antarctic area northward; they also greatly impacted tropical to subtropical oceanic and atmospheric circulation in the North Pacific. The anticyclonic WSCA in the C-BOX was strengthened, thus exciting downwelling oceanic Rossby waves and eventually resulting in equatorward displacement of the NECBL since the early 1990s.

## Data and methods

Monthly SST data of the ERSST (Extended Reconstructed Sea Surface Temperature, version 5) were provided by the NCEI/NOAA (National Centers for Environmental Information/National Oceanic and Atmospheric Administration, https://data.nodc.noaa.gov) with 2° × 2° horizontal resolution since 1854^16^. Monthly sea level height anomalies were from AVISO (Archiving, Validation and Interpretation of Satellite Oceanographic Data, http://www.aviso.altimetry.fr) on a global 0.25° grid since 1993. Monthly wind speed, wind stress, sea level pressure, and geopotential height data were from NCEPr1 (National Centers for Environmental Prediction/National Center for Atmospheric Research reanalysis 1) (https://www.esrl.noaa.gov/psd/data/gridded/data.ncep.reanalysis.html). The products were provided on a global 1.875° grid during the period from 1948 to the present for the NCEPr1. The SAM index^17^ was based on the pressure difference between six stations at 40°S and six stations at 65° S. It was obtained from https://legacy.bas.ac.uk/met/gjma/sam.html.

## Supplementary Information

Supplementary Information.

## Data Availability

Monthly SST data used in the present study were available from the ERSST datasets, https://esgf-node.llnl.gov/search/cmip5/. Monthly sea level height anomalies were available from AVISO datasets, http://www.aviso.altimetry.fr. Monthly wind speed, wind stress, sea level pressure, and geopotential height data were available from the NCEPr1 datasets, https://www.esrl.noaa.gov/psd/data/gridded/data.ncep.reanalysis.html. The SAM index was available from https://legacy.bas.ac.uk/met/gjma/sam.html. The software for the wavelet coherence analysis was provided by A. Grinsted (http://www.glaciology.net/wavelet-coherence).
