# Supplementary material for: Intensified modulation of the Pacific north equatorial current bifurcation by the southern annular mode since the early 1990s
Source: Sci Rep. 2022 Dec 8;12:21210. doi: 10.1038/s41598-022-25661-w (PMC9731944; doi:10.1038/s41598-022-25661-w)
Supplement: Supplementary file 1 — Supplementary Information. [file 41598_2022_25661_MOESM1_ESM.doc]

**Supporting Information for**

**Intensified Modulation of the Pacific North Equatorial Current Bifurcation by the Southern Annular Mode since the Early 1990s**

Li-Chiao Wang^1^, Yong-Fu Lin^2^, and Chau-Ron Wu^3,4,*^

*^1^Department of Atmospheric Sciences, National Central University, Taoyuan, Taiwan*

*^2^Department of Earth System Science, University of California Irvine, California, USA*

*^3^Department of Earth Sciences, National Taiwan Normal University, Taipei, Taiwan*

*^4^Research Center for Environmental Changes, Academia Sinica, Taipei, Taiwan*

**Contents of this file**

Figure S1


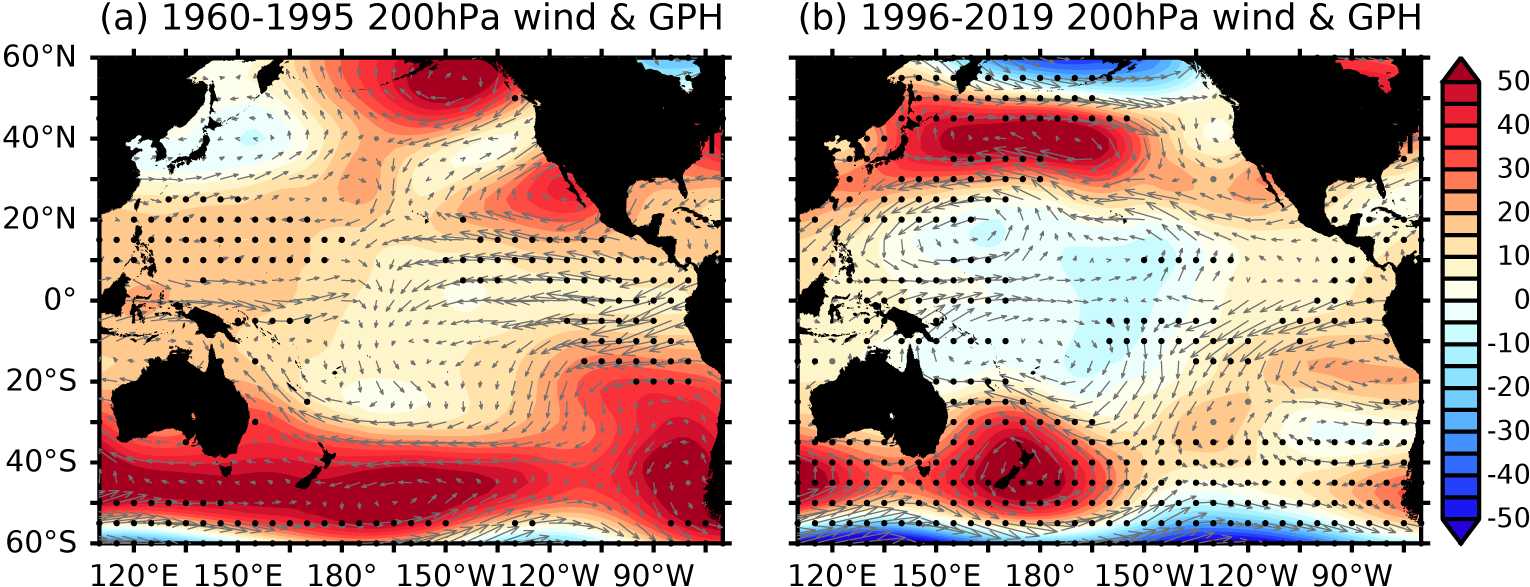


Figure S1. (a) Regression of 15-month running mean of geopotential height (shading, unit in m) and wind anomalies (vectors, unit in m/s) at 200 hPa onto the 15-month running mean of the SAM index during 1960-1995. (b) same as (a), but for 1996-2019. Stippled area in all figures indicates statistically significance at the 95% confidence level determined by a Student’s t-test.
